# Novel technology for automated cleaning of flexible endoscopes

**DOI:** 10.1055/a-2527-4224

**Published:** 2025-03-14

**Authors:** Michelle J. Alfa

**Affiliations:** 1 AlfaMed Consulting, Winnipeg, Canada

**Keywords:** Pancreatobiliary (ERCP/PTCD), Quality and logistical aspects, Quality management, Hygiene

## Abstract

Reprocessing of flexible endoscopes is a multi-stage system with many sequential stages. Errors in any one of the stages can result in microbial contamination that persists in patient ready endoscopes despite full reprocessing. One stage that is especially prone to errors is the manual cleaning of channels and exterior surfaces of flexible endoscopes. This editorial discusses the current factors in manual cleaning that lead to errors in cleaning adequacy. It also reviews novel technologies that provide improvements in cleaning of flexible endoscope channels.



This editorial comments on the important insights presented in the recent publication by van der Ploeg 1 regarding a new novel technology that provides automated cleaning for flexible duodenoscopes. The key objective of this clinical study was to determine if the automated cleaning technology could reduce contamination of fully reprocessed endoscopes. This is an important objective because recent clinical studies indicate that contamination with actionable microorganisms in fully reprocessed flexible duodenoscopes with fixed tips varies in different countries from 4.1% for duodenoscopes in the United States 2 to 12.6% in Europe 3 . Furthermore, Pineau’s study 3 in France provides the largest database for routine culture results for all types of flexible endoscopes spanning 2004 to 2021 from 490 private or public healthcare facilities. This database demonstrated ongoing contamination that varied depending on type of flexible endoscope with an overall actionable rate of 19.7% in 2004 and 13.0% in 2021. Despite the design change to disposable endcaps, the recent clinical study in the Netherlands 4 reported that contamination of fully reprocessed duodenoscope channels remained high at 18.9%. Beyond endoscope design changes, there is a need to improve compliance with manufacturer instructions for use (MIFU) at all stages of flexible endoscope reprocessing.

The overall reprocessing stages of patient-used flexible endoscopes are shown in Fig. 1 . The focus of this editorial is the cleaning stage, but it is imperative to recognize that optimization of ALL stages in reprocessing is needed for an effective outcome. A recent survey by Sivek et. al. 5 indicated that of all the stages, the reprocessing personnel found the manual cleaning stage to be the most challenging stage of endoscope reprocessing. Indeed, van der Ploeg et al. 4 identified that when the manual cleaning stage for patient-used duodenoscopes was completed in 5 minutes or less, there was a statistically significant association with higher odds of microbial contamination with gut and/or oral cavity organisms after high-level disinfection (HLD). In addition to duration of the cleaning stage, it is clear that friction during manual cleaning of all inner-diameter flexible endoscope channels is crucial to ensure that patient residuals and buildup biofilm are physically removed 6 7 8 .

**Fig. 1:**
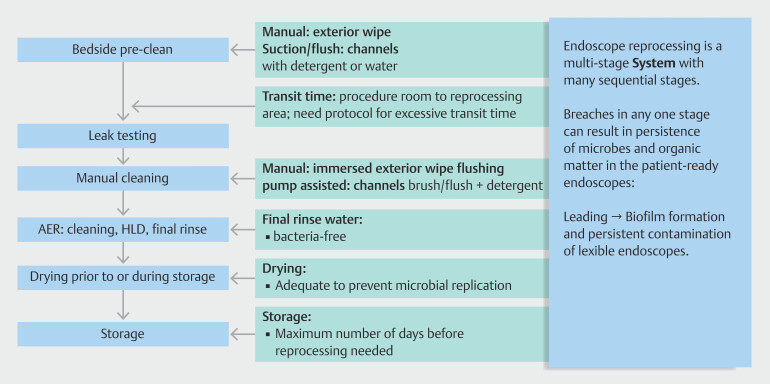
Flexible endoscope reprocessing: A multi-stage system.

Recent publications have confirmed that the air/water channels are the most difficult to dry after full reprocessing 9 , increasing risk of biofilm formation. Furthermore, even when the air/water channels in the insertion tube of patient-used gastroscopes are replaced, they rapidly redevelop biofilm 10 . This is not a surprising finding because the majority of the current MIFUs recommend flushing detergent solutions through the endoscope channels, and although the MIFUs also require bristle brushing of the instrument and suction channels, there is no friction required for the air/water channels.

An urgent need for endoscope reprocessing is integration of efficient friction for all inner diameter size of channels in flexible endoscopes. The recent publication by van der Ploeg et. al. 1 provides data from a clinical evaluation of aquaTyphoon (AT), which is a novel technology for replacing the manual cleaning stage of endoscope reprocessing with an automated process based on turbulent fluid flow. Turbulent fluid flow has been shown to provide friction equivalent to bristle brushes in the suction/instrument channel and superior friction compared with fluid-only flushing in the air/water channels 11 . The van der Ploeg et. al. 1 prospective clinical study compared contamination rates for AT cleaning with retrospective contamination rates for traditional manual cleaning of eight Pentax ED34-i10T2 duodenoscopes (single-use endcaps). Culture interpretation was based on European guidelines whereby MGO (microorganisms of gut or oral origin) and AM20 (any microorganism over 20 CFU) were considered actionable. The objective of this study was to show non-inferiority of AT-processed duodenoscopes to those that received conventional manual cleaning 1 . The AT process utilizes turbulent fluid flow of tap water (without added detergent or single-use brushes) for a 5-minute cleaning cycle of all patient-used duodenoscope channels. The exterior surface is cleaned using an AquaJET sprayer that also does not utilize detergent or cloth wipes. The endoscope is not immersed in water during AT cleaning of channels or AquaJET cleaning of the exterior.

The results of the van der Ploeg et. al. 1 study indicated that the AT cleaning technology (N=100) reduced overall MGO contamination of patient-ready duodenoscopes from 21.6% to 16% compared with conventional manual cleaning (N = 333). It eliminated contamination with *Pseudomonas aeruginosa* , and *Klebsiella pneumoniae* (key pathogens involved in contaminated duodenoscope outbreaks). Furthermore, *K. oxytoca,* and *Enterobacter cloacae complex* were eliminated whereas contamination rates for *Escherichia coli,* and *Citrobacter koseri* were lowered. However, there was a significant increase in contamination with *Stenotrophomonas maltophilia* from 0.3% to 7.0%. Furthermore, there was no reduction in contamination with AM20. As such, non-inferiority for contamination with MGO, oral flora, or AM20 was not demonstrated, but non-inferiority was achieved for gut bacteria.

The results of this novel AT technology 1 are encouraging in that pivotal gut organisms are eliminated or reduced by AT channel cleaning compared with manual channel cleaning, thereby confirming that improvements in cleaning can reduce risk of contamination of patient-ready duodenoscopes. This study supports results of their previous clinical study, which also showed reduced contamination rates when a novel modified cleaning brush was used for suction and instrument channels 7 . The current AT study 1 extends the concept of improved cleaning by automating the cleaning process for all endoscope channels. However, there are several aspects that require further evaluation.

The AT process utilizes an AquaJET spray to clean the outside of the duodenoscopes using tap water without immersion and without detergent. Potable water is acceptable for cleaning of flexible endoscopes 12 but non-immersion of patient-used duodenoscopes during the cleaning process raises several issues. A key concept for immersing endoscopes in detergent solution during manual cleaning is prevention of aerosol generation that could contaminate the environment and/or reprocessing staff. AAMI ST91 12 states: “Sinks should be deep enough to allow complete immersion of the endoscope to minimize aerosolization.” This is particularly important to prevent creation of reservoirs of multi-antibiotic-resistant gut organisms in the reprocessing environment and/or sink drains and to prevent exposure to reprocessing personnel. Use of the AquaJET spray alone for cleaning the external components of patient-used endoscopes would generate aerosolizes of any patient organic and microbial contaminants on the exterior of the patient-used duodenoscope. The authors do comment on this in the discussion, suggesting that immersion in a detergent solution during the external duodenoscope cleaning phase could improve effectiveness of cleaning.

Is 5 minutes of AT cleaning of endoscope channels sufficient or could a longer cleaning time improve results? Data presented for 5 minutes of AT cleaning of duodenoscope channels clearly confirm that not all MGOs or AM20 microbial levels are eliminated despite going through HLD after the AT cleaning process 1 . In the methods section, the authors indicate that prior to initiating the clinical study, all the duodenoscopes underwent four sequential AT cycles without intermittent patient use. Testing after this initial process showed the duodenoscopes were culture-negative. This raises the possibility that a longer AT cleaning cycle for each patient-used duodenoscope may provide further reduction of MGO and AM20 culture results in patient-ready duodenoscopes.

The increase in *S. maltophilia* from the duodenoscope channel cultures after full reprocessing suggests a potential environmental source. The authors state that the sink drain at the location of AT use was culture-positive for *S. maltophilia* and possible splashes from the drain may have contaminated the endoscopes during AT cleaning. This suspected open drain source supports the value of plugging the drain and using full immersion in the sink during the cleaning process, followed by cleaning and disinfection of the sink between each scope cleaning. The authors also indicate that the AT is a moist environment that would be prone to biofilm development. They recommend that the AT be regularly monitored for contamination. However, ST91 12 states that when using flushing pumps for the manual cleaning stage; “…the connection tubing and equipment should be cleaned and disinfected according to the manufacturer’s written IFU”. There was no indication whether the manufacturer provided instructions for use for this process.

One of the major limitations of this study is that there were no data regarding residual organic residues post AT cleaning. As such, it is unclear if this novel AT cleaning technology provides improved cleaning of patient-used duodenoscopes or not. Although the authors clearly state that the objective was to determine the effect of improved cleaning on persistence of MGO and AM20 contamination in patient-ready duodenoscopes, it seems that an opportunity was missed to also evaluate whether the AT cleaning achieved accepted cleaning benchmarks for protein, hemoglobin, carbohydrate, and total organic carbon 12 13 . Because of this limitation, the cleaning efficacy of the AT novel technology for narrow channels (e.g. air/water channels) and wider channels (e.g. suction/instrument channels) cannot be compared with data from other novel cleaning technologies, such as the automated endoscope channel cleaner (AECC) 8 . The AECC showed effective removal of cyclic buildup biofilm, as well as protein, and TOC from 3.7-mm and 1.4-mm inner diameter channels 8 that met cleaning benchmarks in current guidelines 12 13 . This type of information is also important to assess efficacy for external cleaning by the AquaJET because it does not include immersion or use of detergent. In addition, it is crucial that samples for culture and for detection of residuals are taken from the air/water channel in addition to the other channels within flexible endoscopes to ensure adequacy of cleaning and disinfection of these very narrow channels.

In summary, the study by van der Ploeg et. al. 1 is an excellent clinical evaluation of the AT technology for cleaning of patient-used duodenoscope channels. It provides data confirming that improved cleaning of duodenoscope channels can reduce risk of gut organisms contaminating patient-ready endoscopes. In addition, it also identifies areas that need further research including: 1) determining optimal duration of the AT channel cleaning cycle that achieves recommended cleaning endpoints for organic residues; 2) sampling of air/water channels in addition to suction/instrument channels to provide data supporting where contamination post HLD remains and whether adequate cleaning was achieved for the narrowest endoscope channels in addition to the wider inner channel diameter channels; and 3) providing data establishing whether non-immersion technologies such as the AquaJET are able to adequately clean external components of endoscopes without generating aerosols of patient-derived microorganisms from the endoscope or environmental organisms from the sink drain.

## References

[1] Van der Ploeg K Severin J Vos MC Evaluation of a novel water-based automated endoscope Cleaning process compared to conventional manual cleaning for reducing duodenoscope contamination Endosc Int Open 2025 10.1055/a-2536-8061 PMC11922170 40109309

[2] Okamoto N Sczaniecka A Hirano M A prospective, multicenter, clinical study of duodenoscope contamination after reprocessing Infect Control Hosp Epidemiol 2022 43 1901 1909 10.1017/ice.2021.525 35300743 PMC9753065

[3] Pineau L Endoscope reprocessing: Retrospective analysis of 90,311 samples Endosc Int Open 2023 11 E247 E257 10.1055/a-1991-1391 36937825 PMC10023244

[4] Van der Ploeg K Vos MC Erler NS Impact of duodenoscope reprocessing factors on duodenoscope contamination: A retrospective observational study J Hosp Infect 2024 154 88 94 10.1016/j.jhin.2024.09.018 39389430

[5] Sivek A Davis J Tremoulet P Healthcare worker feedback on duodenoscope reprocessing workflow and ergonomics Am J Infect Control 2022 50 1038 1048 10.1016/j.ajic.2022.01.012 35108583

[6] Alfa MJ Singh H Nugent Z et al. Simulated-use polytetrafluorethylene biofilm model: repeated rounds of complete reprocessing lead to accumulation of organic debris and viable bacteria Infect Control Hosp Epidemiol 2017 38 1284 1290 10.1017/ice.2017.215 29039290

[7] Van der Ploeg K Haanappel CP Voor in ’t holt AF Effect of novel endoscope cleaning brush on duodenoscope contamination Endoscopy 2024 56 198 204 37848074 10.1055/a-2193-4481 PMC11583001

[8] Moshkanbaryans L Shah V Tan LY Comparison of two endoscope channel cleaning approaches to remove cyclic build-up biofilm J Hosp Infect 2024 150 91 95 10.1016/j.jhin.2024.05.014 38830542

[9] Yassin M Clifford A Dixon H How effective are the alcohol flush and drying cycles of automated endoscope Reprocessors? Stripped endoscope model Am J Infect Control 2023 51 527 532 10.1016/j.ajic.2023.02.008 36842713

[10] Primo MGB Tipple AFG de Melo Costa D Biofilm accumulation in new flexible gastroscope channels in clinical use Infect Control Hosp Epidemiol 2022 43 174 180 34128460 10.1017/ice.2021.99

[11] Sohn SY Alfa MJ Lai R Turbulent fluid flow is a novel closed-system sample extraction method for flexible endoscope channels of various inner diameters J Microbiol Methods 2020 168 105782 31758953 10.1016/j.mimet.2019.105782 PMC6939870

[12] ANSI/AAMI ST91:2021 Flexible and semi-rigid endoscope processing in health care facilities https://www.aami.org/st91

[13] Z15883–5-09 Washer-disinfectors – Part 5: Test soils and methods for demonstrating cleaning efficacy https://webstore.ansi.org/standards/csa/csaz1588309r2019–2448601

